# Extirpation of an Inverted Uterus
*North of England Medical and Surgical Journal, No. II


**Published:** 1831-01-01

**Authors:** 


### EXTIRPATION OF AN INVERTED UTERUS.*

DR. SYMONDS, of Oxford, has published a case of this description in the last number of our North of England contemporary; the result of the operation was fatal.

*Case.* Mrs. Tidmarsh, æt. 18, was delivered at the full time, about two years and a half ago, of a living child; the labour was lingering, and the placenta long detained, and removed with great violence. In a day or two fever of a low type came on, and more or less uterine hæmorrhage continued for the space of nine months, when she was removed to Oxford. She was very pale and weak; the vaginal discharge was exsanguious, mostly liquid, but occasionally mixed with coagula, and she had now and then bearing down pains. On examination there was found in the vagina a tumour about two inches and a half in length, and an inch and a quarter in its transverse diameter, broadest at its inferior extremity, and slightly tapering towards the point of its attachment—firm and incompressible in texture—smooth and equable on its surface—devoid of sensibility when pressed or irritated. The os uteri embraced its upper part, and was without its usual tuberculated feel. The origin of the tumour was evidently above the mouth of the womb; the finger was stopped in its progress at the pubic and lateral parts, but proceeded higher up posteriorly. Dr. Symonds' father affirmed that the cul-de-sac was entire towards the sacrum, as well as in the other directions.

It was doubtful whether this was a case of polypus or of inverted uterus. The patient improving in some degree, in health, went back into the country, and did not return to Oxford till the latter end of last October. The discharge from the vagina had continued more or less during the interval, having

* North of England Medical and Surgical Journal, No. II.

sometimes been greater, sometimes less; sometimes sanguineous, sometimes whitish. Her general appearance was much the same, excepting that she was not quite so thin. The tumour was decidedly lower down, and so was the os tincæ also. The lips of the latter were thin, and the point of the finger was prevented from penetrating higher than a quarter of an inch, by the unequivocal continuity between the neck of the tumour and the inner surface of the mouth of the womb. It was now decided that the case was one of inversio uteri. At the wish of the patient and her husband, it was determined to remove the tumour by the ligature.

The rectum having been well cleared out by an enema, Mr. Webb, in the presence of Dr. Symonds' father, Mr. Price, and himself, surrounded the neck of the tumour within the os tincæ with a piece of strong whipcord, by means of Dr. Gooch's convenient instrument. The patient experienced little pain, but was rather irritable after the operation, and had a dose of the liq. op. sedativ. All went on tolerably well, the pulse being frequent and irritable, till the third day, when the ligature was tightened. In two or three hours pain came on, and was subdued by opium. The discharge was dreadfully fetid. The ligature was tightened every other day, and on the 15th day it came away and the tumour was extracted. It proved to be an inverted uterus. At the narrow extremity was a basin-shaped cavity, lined with a smooth shining membrane, evidently a part of the peritoneal coat, and at the broader end, which was the inverted fundus, were perceived the orifices of the fallopian tubes. For three or four days there were no marked unfavourable symptoms, yet the pulse was seldom or never below 100; there was faintness on attempting to sit up, and disinclination to solid food. On the fifth day matters were worse, the face was sallow and tumid, there was a peculiar restless moving of the eye, the pulse was hurried, vibratory and small, the breathing was anxious, and the belly was swollen. She had taken a moderate quantity of wine for some days, and an anodyne was now given. In the night there came on all the symptoms of acute peritonitis, rigors followed, and in the middle of the 6th day the poor woman died.

*Dissection.* There was nearly a quart of pus in the cavity of the peritoneum, with adhesions of the omentum to the bladder. There was no evident vascularity, except in the pelvic portion of the peritoneum. There was a free and open passage between the vagina and abdominal cavity; the aperture circular, capable of admitting the finger, and consisting of the ring of the os uteri with about three lines of the cervix. Close upon it were the ovaries, of natural appearance, and remains of the fallopian tubes. The liver was of palish colour—the spleen enlarged—and the heart hypertrophied.

We have, on a former occasion, related the case which occurred to Dr. Gooch, but as it is very short, and acquires additional value by being compared with the preceding, we cannot do better than insert it in this place.

"The first time," says Dr. Gooch, "that I saw the patient was in consultation with Dr. Clarke and Dr. Henry Davies; she had been delivered some months before at St. Omer, and immediately after the removal of the placenta, which had been extracted with some violence, a tumour had been felt projecting from the uterus into the vagina, since which she had not only had no hæmorrhage, but had not even ordinary menstruation. When we examined the tumour, we found it about the size of a small apple with a smooth surface, a somewhat narrow stalk, which was completely encircled by the orifice of the uterus exactly like a polypus, but its quick sensibility to touch, and the circumstances under which it made its first appearance, inclined us to believe that it was an inverted uterus, and not to recommend its removal, particularly as she was losing no blood, and her health was sustaining no injury from it. She returned to the continent, and I did not see her again for two years, when she again came to London, to place herself under the care of Dr.

Granville, who had recommended her to submit to an attempt to revert it, and I now saw her in consultation with the Doctor. Since my former interview with her, she had become subject to frequent and profuse hæmorrhages, which had bleached her face and broken her health, and it now became an urgent object to afford her relief even at some risk. We agreed, therefore, that the attempt should be made to revert the tumour, but if this failed, which appeared most likely, we proposed to her husband the removal of the tumour by ligature, stating to him that such an operation had been done successfully, but that it was attended with considerable risk. This, both he and the patient were willing to incur; the attempt at reduction failed, but before applying the ligature, her former attendants, Dr. Clarke and Dr. Henry Davies were consulted, and all of us agreeing to recommend the operation, the ligature was applied by Dr. Clarke; it was tightened every other day, and each day occasioned so much pain as to require an opiate to quiet it. At length, on the fourteenth day, both instrument and tumour came away; there were times when I had a strong suspicion that it was a polypus, but a sight of the tumour proved that it was the fundus of the uterus, for it was a hollow cup, the size of a small apple, in the cavity of which could be seen the Fallopian Tubes. Excepting the pain, and some vomiting, the patient had no bad symptoms during the cure, and several months afterwards her husband called on me to say she was quite well."

Dr. Symonds appends some long but not inapposite remarks on the diagnosis of inverted uterus. They do not admit of abridgment, and are too diffuse to be transferred entire to our pages. The concluding observations, however, are worthy of attention. We should mention that the patient was found to have taken large quantities of brandy privately after the operation.

"On the whole, if on a future occasion I should discover in the vagina of a woman, who, as far as could be collected, had suffered violence in the extraction of the placenta, a bulbiform tumour, encircled by the margin of the os uteri, and sensible, I should feel satisfied that it was an inverted uterus; but even though it were insensible, if it remained unaltered for several months, and a cul-de-sac were perceptible between that surface of the tumour which the os uteri encloses, and the lips of the latter, as far as I can see at present, I should likewise feel no hesitation in pronouncing it to be of that nature, though at the same time it would be rashness to say, that no other complications could occur to embarrass the judgment.

With regard to the operation, no one will hesitate to think that the operation was warrantable. Successful cases have often occurred besides the one so frequently referred to above; and the circumstances of the patient's health were such, that the choice seemed death from exhaustion, or the risk of extirpation. Of course in Mrs. T.'s case, as in many others which demand serious operations, the very condition of health which renders the interference of art so desirable, at the same time renders the attempt less likely to be successful. The only remark which offers itself on the actual operation, is whether it would not be adviseable in another instance to fix the ligature at a lower point of the tumour, so that the peritoneal coat might not be included. This was suggested to me by the examination of the tumour, in which I observed that the depth of the cavity lined by that membrane, was not more than a third of an inch.

It is deserving of notice that no pain was felt, till some little time after the application, or successive tightenings of the ligature, and which I can only account for, by supposing that the pain arose not so much from compression of the uterine substance, as from that of the surfaces of the peritoneal covering, and the consequent inducement or aggravation of inflammation in that part. That inflammation spread extensively over the peritoneum, was abundantly testified by the dissection, but its progress, (as is not unusual with peritonitis) was very insidious.

The only violent attack of pain, and pain diffused over the whole abdomen, which took place a few hours before death, must, I should conjecture, be attributed to the sudden escape of pus which had been before confined in the pelvic, and hypogastric regions, by the artificial sacs formed of agglutinated folds of intestines and omentum."

